# Probiotic Species in the Modulation of Gut Microbiota: An Overview

**DOI:** 10.1155/2018/9478630

**Published:** 2018-05-08

**Authors:** Md. Abul Kalam Azad, Manobendro Sarker, Tiejun Li, Jie Yin

**Affiliations:** ^1^Key Laboratory of Agro-Ecological Processes in Subtropical Region, Institute of Subtropical Agriculture, Chinese Academy of Sciences, National Engineering Laboratory for Pollution Control and Waste Utilization in Livestock and Poultry Production, Changsha, Hunan 410125, China; ^2^University of Chinese Academy of Sciences, Beijing 100049, China; ^3^Department of Food Engineering and Technology, State University of Bangladesh, Dhaka 1205, Bangladesh

## Abstract

Probiotics are microbial strains that are beneficial to health, and their potential has recently led to a significant increase in research interest in their use to modulate the gut microbiota. The animal gut is a complex ecosystem of host cells, microbiota, and available nutrients, and the microbiota prevents several degenerative diseases in humans and animals via immunomodulation. The gut microbiota and its influence on human nutrition, metabolism, physiology, and immunity are addressed, and several probiotic species and strains are discussed to improve the understanding of modulation of gut microbiota. This paper provides a broad review of several* Lactobacillus* spp.,* Bifidobacterium* spp., and other coliform bacteria as the most promising probiotic species and their role in the prevention of degenerative diseases, such as obesity, diabetes, cancer, cardiovascular diseases, malignancy, liver disease, and inflammatory bowel disease. This review also discusses a recent study of* Saccharomyces* spp. in which inflammation was prevented by promotion of proinflammatory immune function via the production of short-chain fatty acids. A summary of gut microbiota alteration with future perspectives is also provided.

## 1. Introduction

Alteration of the gut microbiota with probiotic species is very prominent in human and animal disease treatment. The potential of probiotic species has recently motivated researchers to examine the production of probiotic foods and the modulation of the gut microbiota. The importance of consumption of probiotic foods with a specific mix of bacteria has been widely studied since the beginning of the 20th century, and yogurt has drawn attention to maintaining good health via development of the digestive system and the prevention of various degenerative diseases [1–3].

The word “probiotic” comes from Greek and means “for life.” In 1954, Ferdinand Vergin conceived the term “probiotic” in an article entitled “Anti-und Probiotika,” in which several microorganisms were studied to make a list of useful bacteria and to determine the detrimental effects of antibacterial agents and antibiotics on the intestinal microbiota [4]. A few years later, Lilly and Stillwell described probiotics as beneficial microorganisms that exert growth-promoting factors for other microorganisms [5]. The term “probiotics” has been modified over time and with research into their application and clinical trials in various human and animal models. According to the Food and Agriculture Organization (FAO) and the World Health Organization (WHO), probiotics are live strains of microorganisms that confer health benefits upon the host when administrated in adequate amounts [6], and this definition is followed by the International Scientific Association for Probiotics and Prebiotics (ISAPP) [7, 8]. However, researchers continue to develop new probiotic species, even though probiotic species have long been used for human health improvement. Most probiotic products today are developed with* Bifidobacteria*,* Lactobacilli*, and other lactic acid bacteria, such as* Lactococci* and* Streptococci*. Other promising probiotic strains include the bacterial genera* Bacillus*,* Escherichia*, and* Propionibacterium* and some other yeast genera, mainly* Saccharomyces*. Probiotics are usually considered to be safe for human health with limited adverse effects [9]. Several species and strains of* Lactobacilli*, including* Lactobacillus acidophilus*,* Lactobacillus casei*,* Lactobacillus rhamnosus*, and* Lactobacillus helveticus*, have been extensively studied in the prevention of human and animal diseases. These probiotic species are able to change the population of microorganisms in the gut microbiota and control the functioning of the ecosystem of gut microbiota. In earlier studies, considerable evidence of clinical trials of probiotics in animal and human models has reported suitability for the treatment of a variety of diseases, and this number continues to grow.

The human gut is a complex ecosystem in which nutrients, the microbiota, and host cells interact extensively. The relationships between these microorganisms and host cells were long considered only from a pathogenic point of view because toxins invade the gut mucosa and translocate, disseminate, and cause systemic infections [10]. However, no attention was paid to the majority of gut microorganisms and their relationship with host health. Several studies have reported beneficial interactions between the commensal microbiota and the human body and have indicated that the microbiota acts as a real partner. A deeper understanding of the gut microbiota and its role is necessary for future healthcare strategies. In this regard, extensive study of the potential use of selected probiotic bacteria species and their strains is desperately needed for the prevention and treatment of numerous human and animal diseases [11–14].

The relationship between health and the composition of the gut microbiota has raised interest in the modulation of the gut microbiota by administration of probiotic species for the prevention of some diseases in humans and animals. This review focuses on the gut microbiota and several probiotic species that have been extensively studied in the modulation of the gut microbiota and prevention of degenerative diseases.

## 2. Gut Microbiota

The term “gut microbiota” was first introduced to the scientific community by Joshua Lederberg who called it “the ecological community of commensal, symbiotic, and pathogenic microorganism that literally share our body space and have been all but ignored as determinants of health diseases” [31]. The human body consists of trillions of microbes, mostly within the gastrointestinal tract (i.e., the small intestine and colon). Using a 70 kg man as a reference, 3.8 × 10^13^ microbes are reported to have a total weight of 0.2 kg [32]. The gut microbiota can ferment nondigestible carbohydrates, which are well known as prebiotics, including fructooligosaccharide, oligofructose, inulin, galactose, and xylose, that contain oligosaccharides to fulfill energy requirements. The microbes in the host body have a significant influence on the metabolism, physiology, and immune development and function, whereas symbiotic functions include the synthesis of vitamins, protection from pathogenic colonization as a regulatory immune system via modulation of gastrointestinal hormone release function, and regulation of brain behavior in terms of neuronal signaling [33–38]. The improvement of culture-independent and molecular high-throughput techniques favor the identification of previously unknown bacteria, which would provide novel insights into the functional capacity and compositional diversity of some of the fecal microbiota. In addition, several studies have suggested that disorders such as colorectal cancer, inflammatory bowel disease (IBD), alcoholic and nonalcoholic fatty liver diseases, obesity, type 2 diabetes, oxidative stress–related disease, and immune-mediated diseases are associated with disease-specific dibiotic of altered microbiota compositions [15, 39–43]. Modification of the gut microbiota has thus gained more attention as a potential treatment for several diseases in humans and animals.

## 3. Modulation of Gut Microbiota and Probiotic Species

The gut microbiota includes bacteria, fungi, archaea, protozoa, and viruses that interact with the host and each other to affect the host's physiology and health [44]. The gut bacteria play significant roles in human health, including vitamin B synthesis, improvement in digestion, and promotion of angiogenesis and nerve function [45]. In addition, modification of the gut microbiota can be harmful when the gut ecosystem undergoes severe abnormal changes. The bacterial species found in the human gut microbiome include mostly three phyla: Bacteroidetes* (Porphyromonas, Prevotella)*, Firmicutes* (Ruminococcus, Clostridium, *and* Eubacteria)*, and Actinobacteria* (Bifidobacterium)*.* Lactobacilli, Streptococci*, and* Escherichia coli *are found in small numbers in the gut. However, alteration of the gut microbiota composition can lead to multiple diseases in humans and animals [21, 22, 28, 30].

Current evidence supports a link between the activity and composition of the gut microbiota and human health and disease. Furthermore, the gut microbiota composition is likely to affect many organ systems, including the cardiovascular, neural, immune, and metabolic systems. The gut microbiota composition is altered in many disease states, such as cardiovascular disease, cancer, malignancy, type 2 diabetes mellitus, obesity, colitis, asthma, psychiatric disorders, inflammatory disorders, disorders of the gut-brain axis, and numerous immune disorders [15, 40, 41, 46–48]. Modulation of the gut microbiota facilitates a number of health problems; probiotic feeding with a high-fat diet showed alteration of the gut microbiota composition with a decrease in the gram-positive bacteria phyla Firmicutes and Actinobacteria in mice [49]. In contrast, in a mouse model of hyperlipidemia, the probiotic administration of* Lactobacillus *led to significant changes in the microbiota composition, including an increased abundance of Bacteroidetes and Verrucomicrobia and a reduced ratio of Firmicutes [50]. It is evident that probiotic species play important roles in maintaining the gut microbiota ecosystem in humans and animals.

## 4. Bacteria Species

Table 1 presents the most significant results of various studies on the influence of probiotic bacteria species or strains on modulation of the gut microbiota in various models and diseases.

### 4.1. *Lactobacillus*

Most studies of probiotic species in biomedical research have examined the lactic acid bacteria group. In gut microbiota studies,* Lactobacillus *has been reported as the most prominent probiotic from the lactic acid bacteria group. Changes in the composition, diversity, and function of the gut microbiota by probiotic species have been studied using tools and techniques including targeted, culture-dependent methods and metagenomics sequencing. However, a few studies have demonstrated the associations of probiotic species with altered gut microbiota composition. A recent metagenomic analysis of 8-week-old Swiss mice fed a high-fat diet showed that treatment with a probiotic mixture of* Lactobacillus *and* Bifidobacterium *(*L. rhamnosus*,* L. acidophilus*, and* Bifidobacterium bifidum*) significantly altered the composition of the gut microbiota and increased insulin sensitivity. Several authors have reported that mice with a high-fat diet with probiotic species had a lower population of* Firmicutes, Actinobacteria, *and* Bacteroides *than untreated mice [25]. Similar work on obese mice revealed that several* Lactobacillus *spp.*, Bifidobacterium *spp., and other coliform bacteria increased the gut microbiota composition in mice with a high-fat diet treated with various* Lactobacillus *probiotic strains (*L. acidophilus *IMV B-7279,* L. casei *IMV B-7280,* B. animalis *VKL, and* B. animalis *VKB). In addition, the gut microbiota composition of obese mice treated with* L. casei, L. delbrueckii *subsp.* bulgaricus, *and* B. animalis *showed a significant decrease in microscopic fungi [51]. Probiotic species of* Lactobacillus *may improve gastrointestinal barrier function by the proliferation of some harmful bacteria [24, 52]. Intestinal permeability can be achieved with an increase in the intestinal tight-junction protein occludin. After a change in the gut microbiota composition with a probiotic, mice with a high-fat diet were reported to show an increase in the expression of the tight-junction protein, proglucagon mRNA, and reduced intestinal expression of the pattern recognition receptors CD-14 and NOD1. It also leads to a reduction in the circulating level of lipopolysaccharide and an increase in glucagon-like peptide 1. In addition, probiotic-treated mice have shown increases in lipoprotein-lipase-dependent triglyceride storage in adipose tissue and adipocyte triacylglycerol accumulation [25, 53]. Probiotic* Lactobacillus *strains have been found to increase gastrointestinal barrier function by the proliferation of harmful bacteria in nonalcoholic fatty acid liver diseases and IBD [15, 24].

An accumulating body of research on probiotics provides evidence that T regulatory (Treg) cells play a crucial role in maintaining immune homeostasis in many diseases. Treg cells secrete IL-10, IL-17, and IL-22 (anti-inflammatory cytokine) which are important for maintenance of homeostasis [54–56]. Commensal* Lactobacillus *species can restore homeostasis in intestinal disorders and thus play a protective role against inflammatory diseases. A recent study showed that a probiotic species of* Lactobacillus acidophilus (L. acidophilus)* administered for modulation of dextran sulfate sodium-induced colitis restored the balance of inflammatory cytokines and Th17/Treg cells [15]. The authors also reported that* L. acidophilus *suppressed proinflammatory cytokines such as IL-6, tumor necrosis factor-a, and IL-1b in colon tissues. In addition, in vitro treatment by* L. acidophilus *directly induced the production of IL-10 and Treg cells and suppressed the production of IL-17. Similarly, a probiotic strain of* L. acidophilus *isolated from a normal human intestinal tract and orally administrated in mice with dextran sulfate sodium-induced colitis suppressed the colitis-associated response of the IL-23/Th17 axis and reduced the secretion of proinflammatory cytokines [17]. Furthermore, based on Treg cell modulation and Th17-biased immune response in regulatory cytokines, the probiotic strain of* Lactobacillus *spp. showed beneficial effects in preventing cancer and intestinal inflammation [16, 19, 57, 58]. Similarly, a probiotic strain of* L. plantarum *TN8 reduced the proinflammatory cytokine expression and also regulated the intestinal immune system of Wistar rats with trinitrobenzene sulfuric acid-induced colitis [59]. The same probiotic strains* (L. plantarum TN8)* also showed anti-inflammatory properties by inducing production of IL-10 and a small amount of IL-12 cytokines [60].

### 4.2. *Bifidobacterium*

*Bifidobacterium *is important in gut microbiota studies and has long been used as a probiotic to alleviate various diseases by changing the gut microbiota composition. Like other* Lactobacillus*,* Bifidobacterium *can also inhibit harmful bacteria, improve gastrointestinal barrier function, and suppress proinflammatory cytokines [24]. Recent studies have demonstrated that* Bifidobacterium* alters the function of dendritic cells to regulate the intestinal immune homeostasis to harmless antigens and bacteria or initiate protective measures against pathogens. It also has the potential to control various intestinal diseases, like IBD, cancer, and allergies [61–63]. The probiotic* Bifidobacterium *has shown metabolic capacity in gut bacteria and can increase the proportion of beneficial bacteria in the gut microbiota by cross-feeding. According to Turroni et al. [64],* Bifidobacterium bifidum *significantly increased metabolic activity when cocultured with* Bifidobacterium breve*. This coculture of probiotic bacteria affected the metabolic shift in the gut microbiota by increasing the production of short-chain fatty acids rather than by changing the gut microbiota composition. Colonic mucus is a physical barrier that consists of gut microbiota and is maintained by an extensively glycosylated mucin-2 network.* In vivo*, the ability of bacteria-sized beads to penetrate the mucus layer was greater in mice fed a Western-style diet than in chow-fed mice, which indicated slower mucus growth in the mice fed a Western-style diet due to host metabolic factors. It is worth noting that the abundance of* Firmicutes* increased and that of* Bacteroidetes *and* Actinobacteria *were reduced in the colonic lumen of mice fed a Western-style diet. A study with probiotic treatment showed that* Bifidobacterium longum *NCC 2705* (B. longum)* prevented mucus production [65]. Moreover,* Bifidobacterium *exerts a positive effect via hormonal signaling in the gut-brain microbiome axis to improve memory function, including brain-derived neurotropic factor and N-methyl-D-aspartate receptor expression. It has been reported that a combination of* Lactobacilli *and* Bifidobacterium *decreased acute stress and depression [66, 67]. However, the understanding of the molecular mechanism is beyond the scope of this study.

### 4.3. Other Bacteria Species

Like other probiotic species,* Escherichia coli*, a gram-negative bacterium in the* Enterobacteriaceae *family, is a well-known probiotic strain with some beneficial effects on gut microbiota homeostasis. The nonpathogenic strain* Escherichia coli Nissle *(EcN) is one of the most used probiotic strains in gut microbiota homeostasis. It has been shown that EcN can stimulate the production of human *β*-defensin 2, which can protect the mucosal barrier against adhesion and invasion by pathogenic commensals [68, 69]. In addition, several in vivo and in vitro studies have shown that EcN has a protective function against* Salmonella*,* Shigella*,* Candida*, and some other invasive commensals and may restore damaged epithelium by modulation of tight-junction and zonula occludens proteins [70]. However, outer membrane vesicles (OMVs) released by gram-negative bacteria play a vital role in the signaling process of the intestinal gut mucosa. The release of OMVs begins a mechanism to deliver some active compounds and microbial proteins to the host body without intercellular contact. It was recently demonstrated that OMVs trigger the host immune and defense responses of the probiotic strain EcN, which entered intestinal cells via clathrin-mediated endocytosis. In fact, in vitro and ex vivo studies have demonstrated expression of antimicrobial peptides and modulation of the cytokine/chemokine response of gut epithelial and gut immune cells when the probiotic strain EcN induced OMVs. Moreover, these OMVs promote the upregulation of the tight-junction proteins of zonula occludens and claudin-14, but down-regulation of claudin-2 reduces gut permeability and supports intestinal barrier functions in intestinal epithelial cell lines [71, 72]. Finally, the probiotic strain EcN is also involved in the intestinal microbiota immune response, including macrophages, epithelial cells, dendritic cells, and upregulation of proinflammatory cytokines (IL-6, IL-8, and IL-1*β*) [71].

*Enterococcus *are gram-positive bacteria in the lactic acid bacteria family. Some strains of* Enterococcus *exert antibiotic-induced dysbiosis and act as antitumor or anticancer agents and modulate the immune system. It has been found that culture of* E. faecium* strain from human intestinal epithelium increased the bactericidal effects against enteroaggregative* E. coli, *membrane damage, and cell lysis [73, 74]. Fusco et al. [74] characterized intestinal cytokine expression in epithelial cells and reported that intestinal cytokines play a key role in the host inflammatory response to damage by* Salmonella typhimurium*. It has been revealed that* E. faecium* increases the expression of proinflammatory and anti-inflammatory cytokines without appearing as a pathogen. Furthermore,* E. hirae *exerts the gut epithelial barrier function by inducing Th17 [30].

*Saccharomyces* is well-known nonpathogenic selective probiotic yeast that has been used commercially in the production of probiotic foods. Over the past few decades,* S. cerevisiae* and* S. boulardii* have demonstrated extensive promise as a probiotic treatment [28]. Several studies have demonstrated that* S. cerevisiae* and* S. boulardii* were associated with an increased proportion of* Bacteroidetes *in the gut microbiota composition and decreased the relative abundance of* Firmicutes *and* Proteobacteria. *In addition, this yeast has ability to prevent inflammation by promoting proinflammatory immune function and increasing the production of short-chain fatty acids [28, 29, 75, 76].

## 5. Conclusions

Probiotic bacteria species form a reproducible gut microbiota population in various host bodies and diseases. Various probiotic species have been reported to prevent many degenerative diseases, including obesity, diabetes, cancer, cardiovascular disease, malignancy, liver diseases, and IBD. An imbalance of the gut microbiota composition can lead to several diseases. Probiotics have been proved to modulate gut microbiota composition imbalance by increasing bacteria population, gut epithelium barrier function, and cytokine production. Meanwhile, diets and different nutrients have been reported to productively and markedly shape gut microbiota communities [77–83], further studies should be performed to elucidate the metagenomics relationship between alteration of the gut microbiota composition and probiotic species under different diets or nutrients. A well-designed and appropriate experimental model (in vivo, in vitro, or ex vivo) is suggested to provide insights into the gut microbiota composition and potential commensals for host health. Furthermore, the identification of new probiotics and isolation from microbiome and mixture of probiotic species would be a key pathway for future studies to promote host health.

## Figures and Tables

**Table 1:** Some bacterial strains used in gut microbiota modulation.

Bacteria strains	Disease model	Disease	Outcomes	References
*L. acidophilus*	Eight-week-old male C57BL/mice	IBD	↑ IL-10, Treg ↓ IL-6, IL-1*β*, IL-17	[15]
*L. acidophilus *(NCK2025)	Generation of TS4Cre × APC^lox468^ mice	CRC	↑ IL-10, IL-12 ↓ Treg	[16]
*L. acidophilus*	Female BALB/c mice	Crohn's disease	↑ IL-17 ↓ Th17 function, IL-23	[17]
*L. acidophilus*	BALB/c mice	Ulcerative colitis	↑ *Lactobacilli, Bifidobacteria* ↓ *S. aureus*	[18]
*L. casei *BL23	Female C57BL/6 mice	CRC	↑ Th17, Th 22, IL-10, and IL-22 ↓ Treg	[19, 20]
*L. fermentum *FTDC 812	Eight week old BALB/c mice	Hypercholesterolemia	↑ *Lactobacillus*	[21]
*L. johnsonii*	Male C57BL/6 mice	Acute live injury	↑ IL-22, *Lactobacillus*	[22]
*L. plantarum *CCFM10, RS15-3	58 week BALB/c mice	Oxidative stress	↑ *Bacteroidetes, Firmicutes *	[23]
*L. acidophilus* *B. cereus, B. infantis*	Eight week SPE male SD mice	Nonalcoholic fatty liver disease	↑ *E. coli, Enterococcus,* ↓ *Bifidobacteria, Bacteroides, *and* Lactobacillus *	[24]
*L. acidophilus* *L. rhamnosus, B. bifidum*	Eight week C57BL/6 mice	Type 2 diabetes	↑ *Firmicutes, Actinobacteria* ↓ *Bacteroidetes*	[25]
*B. breve *IPLA20004	Human colon	Inflammatory	↑ IL-8, IL-10, IL-12	[26]
*E. coli *Nissle 1917	Male C57BL/6J mice	Chronic inflammation	↑ IL-10, tight-junction ↓ IL-17	[27]
*S. boulardii*	Adult BALB/c mice	Acute liver failure	↑ *Bacteroidetes* ↓ *Firmicutes, Proteobacteria*	[28]
*S. boulardii*	Six week C57BL/6 mice	Type 2 diabetes	↑ *Firmicutes, Proteobacteria, *and* Fibrobacteria*	[29]
*E. hirae*	C57BL/6J mice	Cancer	↑ Th 17 cell response	[30]

## Abbreviations

GIT: Gastrointestinal tract
CVD: Cardiovascular disease
LAB: Lactic acid bacteria
HFD: High-fat diet
LPS: Lipopolysaccharide
Treg: T regulatory cell
IL: Interleukin
Th: T helper cell
DC: Dendritic cell
IBD: Intestinal bowel disease
WSD: Western-style diet
EcN: * Escherichia coli *Nissle
OMVs: Outer membrane vessels.
